# Whole shaft visibility and mechanical performance for active MR catheters using copper-nitinol braided polymer tubes

**DOI:** 10.1186/1532-429X-11-29

**Published:** 2009-08-12

**Authors:** Ozgur Kocaturk, Christina E Saikus, Michael A Guttman, Anthony Z Faranesh, Kanishka Ratnayaka, Cengizhan Ozturk, Elliot R McVeigh, Robert J Lederman

**Affiliations:** 1 Translational Medicine Branch, Division of Intramural Research, National Heart Lung and Blood Institute, National Institutes of Health, Bethesda, MD, USA; 2 Institute of Biomedical Engineering, Bogazici University, Istanbul, Turkey

## Abstract

**Background:**

Catheter visualization and tracking remains a challenge in interventional MR.

Active guidewires can be made conspicuous in "profile" along their whole shaft exploiting metallic core wire and hypotube components that are intrinsic to their mechanical performance. Polymer-based catheters, on the other hand, offer no conductive medium to carry radio frequency waves. We developed a new "active" catheter design for interventional MR with mechanical performance resembling braided X-ray devices. Our 75 cm long hybrid catheter shaft incorporates a wire lattice in a polymer matrix, and contains three distal loop coils in a flexible and torquable 7Fr device. We explored the impact of braid material designs on radiofrequency and mechanical performance.

**Results:**

The incorporation of copper wire into in a superelastic nitinol braided loopless antenna allowed good visualization of the whole shaft (70 cm) *in vitro *and *in vivo *in swine during real-time MR with 1.5 T scanner. Additional distal tip coils enhanced tip visibility. Increasing the copper:nitinol ratio in braiding configurations improved flexibility at the expense of torquability. We found a 16-wire braid of 1:1 copper:nitinol to have the optimum balance of mechanical (trackability, flexibility, torquability) and antenna (signal attenuation) properties. With this configuration, the temperature increase remained less than 2°C during real-time MR within 10 cm horizontal from the isocenter. The design was conspicuous *in vitro *and *in vivo*.

**Conclusion:**

We have engineered a new loopless antenna configuration that imparts interventional MR catheters with satisfactory mechanical and imaging characteristics. This compact loopless antenna design can be generalized to visualize the whole shaft of any general-purpose polymer catheter to perform safe interventional procedures.

## Background

Image-guided catheter intervention using MR remains tantalizing. Real-time imaging [1-5] and patient-handling [6] capabilities have been demonstrated. Nevertheless, few animal demonstrations [7-10] have been translated into human subjects [11-14]. Clinical-grade interventional catheter devices for use during MR, such as catheters and guidewires, remain the most significant obstacle to wider clinical translation. Current non-clinical implementations tend to offer reduced visibility under MR or excessive size and reduced mechanical performance [15,16].

Cardiovascular catheter devices used under X-ray guidance are visible based on their simple attenuation of incident X-ray photons, regardless of configuration or orientation. Comparable devices for operation under MR are more complex. They must be safe for use in the high magnetic field. They must not distort the surrounding magnetic field, which in turn distorts imaging of surrounding anatomy. They must preserve the mechanical properties expected by the interventional operator accustomed to X-ray operation. Finally they must be conspicuous, from the tip throughout the length inserted in the body, for safe procedure conduct during especially complicated procedures.

Approaches to make MR catheter devices conspicuous vary widely. Passive devices rely on intrinsic material characteristics for visibility. Passive devices have used polymer catheters with ferromagnetic [17] or paramagnetic coatings or rings [18], to produce negative or positive contrast, respectively. Other catheters with CO_2 _balloons [19] or filled with more unique contrast agents and multispectral detection such as 19-F [20] and hyperpolarized 13-C [21] have also been explored. The length of these passive devices can be made visible by coating the entire device but this may occupy a large portion of the catheter volume otherwise required for therapeutic purposes, or which may have the potential to leach into the surrounding blood. Off-resonance imaging techniques [22,23] may improve the visibility of such devices, at the expense of imaging target anatomy.

Active devices, which embed antennae, and semi-active devices, which incorporate other electronics, may be more conspicuous than passive devices but have other problems. Often these have limited visibility profiles only part of the device is visualized. They may incorporate conductive hypotubes to provide whole-shaft visibility, at the expense of flexibility or other important mechanical characteristics. Inductively-coupled markers, one semi-active approach, can be incorporated on the distal end of catheters [24,25] and do not require long transmission lines to connect to the scanner. The catheter appearance is then limited to the distal end and may require further optical tuning [26,27] or signal separation [28] to firmly distinguish them from background tissue. Active tip "tracking" (using special non-imaging pulse sequences) or "profiling" (using ordinary imaging) are good for robust device visualization, but traditionally track either the tip or the shaft but not both.

The original tip tracking principle [29] has been adapted to allow tip tracking along with tip orientation [30,31] but the rest of the device location and alignment remains unknown unless it is visualized by an alternate means. Tip profiling could be accomplished with multiple or elongated coils along the length of the device [31,32]. Although the loopless antenna design first described for guidewire design [33], it is typically used for whole-shaft visibility. The loopless antenna usually is a flexible coaxial line whose inner conductor is extended by approximately a quarter wavelength (λ_m_/4, where λ_m _is the wavelength in the body). This antenna design has been used in conjunction with a guide catheter [34] in addition to being modified to create active intramyocardial injection catheters and intravascular needles [35,36]. A coaxial MR compatible metal hypotube and wire configuration must often be used to create loopless antenna design. This can significantly increase the stiffness of the device, particularly as the catheter size increases.

X-ray interventional catheters incorporate a composite polymer-metal design to optimize handling and mechanical performance. Embedding metal braiding within a polymer scaffold provides pushability and torquability to advance and manipulate the device yet retains flexibility required to navigate tortuous paths [37]. This approach has largely been abandoned in iMR devices because of concerns over metal susceptibility artifacts and heating. While widely-used stainless steel braiding may be inappropriate for MR, this composite principle can be used to design antennas that are embedded into the catheter to impart whole-shaft visibility as well as favourable mechanical properties.

In this work, we developed a three channel multi-purpose active catheter prototype using a novel copper and nitinol wire-braided polymer hypotube. Loop coils provide 3 focal image markers on the distal end for tip location and orientation while the loopless antenna with the wire braided polymer tubing provides shaft visibility. The each individual active catheter channels was connected to the 1.5 T MR scanner via specially designed pre-amplifier interface box (Stark, Erlangen, Germany), these features can be displayed in different colors during real-time MR. The device was tested in *in vitro *and *in vivo *animal real-time MR-guided experiments.

## Methods

Catheter prototypes were constructed in the NHLBI core catheter fabrication facility, which has an ISO class 7 clean room.

### Loopless antenna design using braiding layers

**Figure 1:**
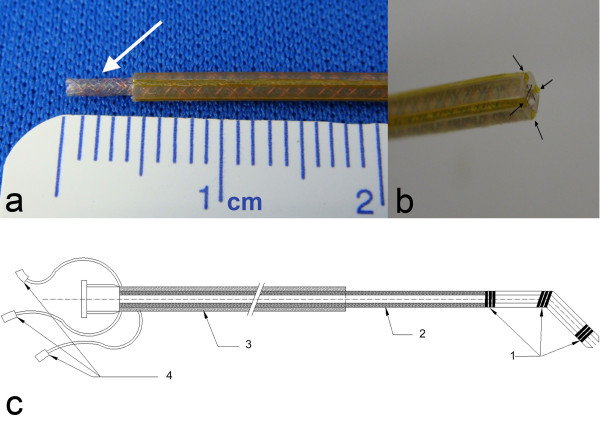
**Braiding setup and catheter shaft structure**. **(a) **Copper and nitinol braiding layer (arrow) exposed after outer *Pebax *layer is removed. **(b) **Four grooves (arrows) are created in the inner polymer shaft to house transmission lines. **(c) **Schematic of a finished 7 Fr guide catheter with a 0.035" guidewire compatible lumen. (1) Three solenoid coils incorporated in the distal shaft for tip profiling (2) Inner nitinol-copper braiding layer used as a core conductor of the loopless antenna (3) Outer braiding layer used as a shield for the loopless antenna. (4) micro-miniature connectors (MMCX).

The design of the loopless antenna was based on coaxial braiding layers that are separated by medical grade thermoplastic elastomer tubing (*Pebax*, Medical Extrusion Technologies, Murrieta, CA) and polyimide tubing (Microlumen, Tampa, FL). Flat (0.003" width, 0.001" wall thickness) and bare copper and nitinol wires were used for each braiding layer (Figure 1a).

To reduce received signal attenuation, the diameter of each braiding layer and the space between two centric braiding layers was adjusted with polymer insulator material (ε_r _= 3.45) to have a characteristic impedance matching the scanner port. The whip length of the dipole antenna was adjusted to have minimum resistance whip length when immersed in a 0.35% saline solution which measured RF electrical properties approximate within the body (ε_r _= 77, σ = 0.6 S/m) by using a network analyzer (Model 4395A, Agilent Technologies, Santa Clara, CA).

### Mechanical evaluation of braiding configurations

Different braiding configurations were prepared with varying nitinol/copper wire ratios. Each braiding configuration was tested in terms of flexibility and torque response, and compared with a braided clinical introducer catheter that has 7Fr inner diameter (*Shuttle*, Cook, Bloomington, IN). Such a near sized late-generation braided catheter or introducer sheath would be used during related X-ray guided interventional procedures.

#### Flexibility test

**Figure 2:**
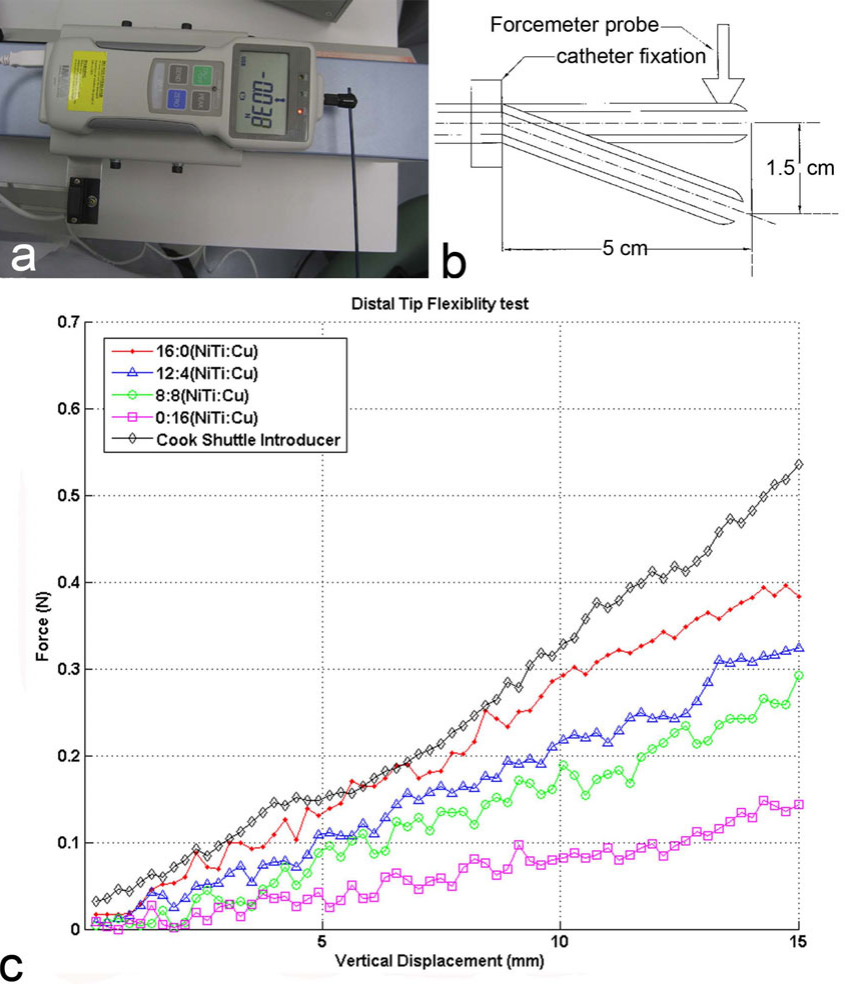
**(a) Flexibility test setup and result**. The catheter distal tip was fixed perpendicular to force meter that was mounted on a motorized stage. **(b) **The schematic representation of tip flexibility measurement setup. **(c) **Distal tip flexibility for different braiding layer configurations. Resistance force at the catheter distal tip was measured while the shaft was bent from 5 cm away until the tip reaches 1.5 cm vertical displacement.

The aim of this test is to compare the force required to deflect the distal tip of the catheter the same amount for different nitinol/copper wire ratio braiding configurations. The catheter distal tip was connected to the force meter in a way that the tip is perpendicular to force meter (Figure 2a). The force meter was mounted onto motorized linear stage that moves at a constant speed. The catheter was fixed 5 cm away from the distal tip to prevent any damage on two solenoid coils at the tip. The force required to deflect the catheter tip up to 1.5 cm was measured using the digital force meter as shown in the schematic representation in Figure 2b.

#### Torquability test

**Figure 3:**
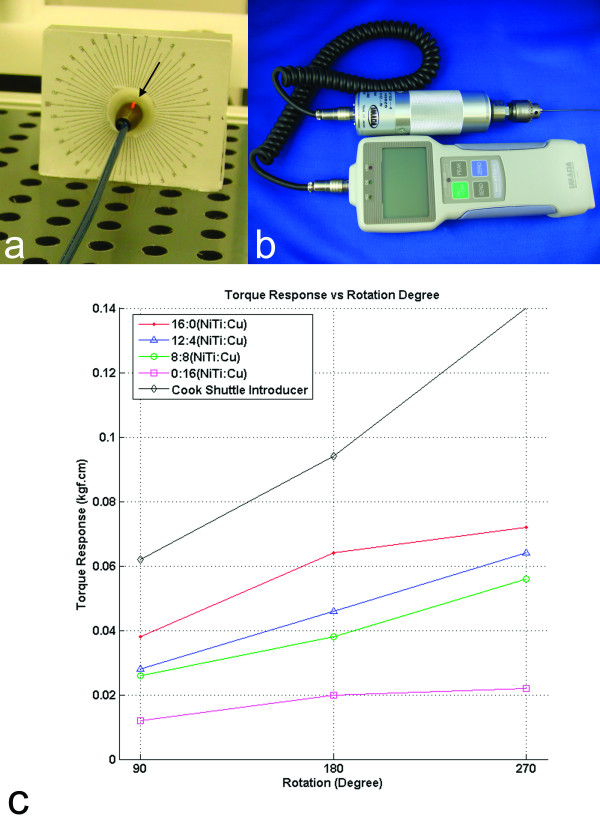
**Torque transmission test fixture setup and test result**. **(a) **The distal shaft is fixed to a freely-rotating collet (arrow) that indicates the angular response to torque. **(b) **A meter on the proximal shaft recorded transmitted torque after 90–270° tip rotation. **(c) **Comparative torquability values for different braiding layer configurations. Higher torque response values indicate greater torquability.

This test demonstrates torque transmission ability from proximal end to the distal end of the catheter shaft. The distal tip of the catheter was attached to a fixed collet that is free to rotate axially. The distal tip of the collet was marked and rotation angle was measured relative to a fixed platform (Figure 3a). The catheter proximal hub was mounted to a digital torque meter (HTG-2, IMADA Inc., Northbrook, IL) and then peak torque values were recorded when the distal tip was rotated 90, 180 and 270 degrees (Figure 3b).

### Catheter construction

*Pebax *custom design extruded tubing was used as the core layer for the active catheter. The core tubing design has four grooves to accommodate micro coaxial coax transmission cables for the distal loop coils. The braiding layer of the core tubing lies under the grooves and forms the core conductor layer of the dipole antenna (Figure 1b).

**Figure 4:**
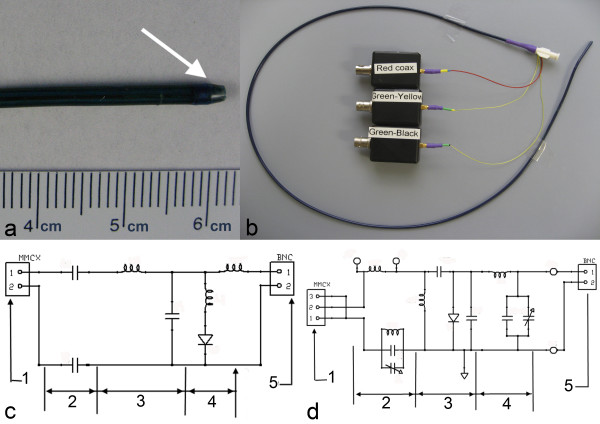
**The active catheter prototype**. **(a) **The atraumatic distal tip of the catheter (arrow). **(b) **Three channel active catheter with matching circuit boxes. **(c) **Schematic of the loop antenna matching/tuning and decoupling circuit a) MMCX (micro BNC) connector b) DC block section c) matching/tuning section d) decoupling section e) BNC connector. **(d) **Schematic of the loopless antenna matching/tuning and decoupling circuit a) MMCX connector b) DC block section c) matching/tuning section d) decoupling section e) BNC connector.

The distal tip contains three loop coils, 2.54 cm apart, to indicate tip position and orientation. The first and third coils share a common transmission line; all used micro coaxial cables that are 100 cm and 0.3 mm in diameter. The second (shield) braiding layer was positioned over these transmission lines, adjusted to minimum resistance length. Both layers were fused together by heating up to 193°C (Figure 1c). Afterwards the distal tip of the catheter was tapered and rounded (Figure 4a). The 7 Fr catheter prototype has three additional tip coils, a 0.035" guidewire-compatible inner lumen and 75 cm overall length. Figure 4b shows the complete device and connectors.

All loop and loopless antenna channels were tuned to Larmour frequency of a 1.5 T MR scanner and matched to 50 Ω in a saline bath (0.35% NaCl, ε_r _= 77, σ = 0.6 S/m) using dedicated matching/detuning circuit boxes. The proximal end of each antenna was connected to dedicated matching and decoupling circuits (Figure 4c, d).

### Visibility performance testing under MR

MR scans were performed at 1.5 T (Magnetom Espree, Siemens Healthcare, Malvern, PA). The active catheter was connected in receive-only mode to the flex coil adaptor scanner using a custom 4-channel pre-amplifier interface box.

#### Visibility *in vitro*

A phantom filled with a 0.35% NaCl solution was used to simulate loading conditions (ε_r _= 77, σ = 0.6 S/m) of an active catheter inserted into a human body. Real time MR imaging was performed using a balanced steady state free precession (SSFP) sequence with slice thickness, 5 mm; repetition time (TR), 3.68 ms; echo time (TE), 1.84 ms; field of view (FOV), 340 mm; matrix, 192 × 108; bandwidth, 700 Hz/pixel. Signal from different catheter channels were reconstructed in color using a customized interactive real-time MR interface [2]: first- and third- distal loop channels, green; second loop channel, red; loopless shaft, blue.

The *in vitro *signal-to-noise ratio (SNR) profile of the active catheter was mapped in normalized SNR units from a single magnitude image [38] using Matlab (Mathworks Inc., Natick, MA). A reproducible noise region of interest was selected, and corrected for the number of active catheter receiving channels [39].

#### Visibility *in vivo*

*In vivo *experiments were conducted on 5 anesthetized Yorkshire pigs (65 ± 11 kg) in protocols approved by the National Heart Lung and Blood Institute Animal Care and Use Committee. A balanced SSFP sequence used the following parameters: TR/TE, 3.72/1.86; flip angle, 60°; slice thickness, 6.0 mm; FOV, 340 mm; matrix, 192 × 108; bandwidth, 797 Hz/pixel. The same custom real-time MR interface was used to colorize each catheter channel separately. From a transfemoral approach, the active catheter was advanced retrograde over a 0.035" guidewire through the aorta under real-time MR guidance.

### Heating test

Heating tests were performed to evaluate the RF induced temperature increase characteristics of the 7 Fr active prototype catheter. We used an acrylic phantom (41.9 (w) × 71.1 cm (l) × 15 cm (d)) based on the ASTM standard [40]. Acrylic posts and pegboard were used to stabilize the active catheter position for each configuration during heating experiments. The phantom was filled with a polyacrylic acid gel (5.85 g/L polyacrylic acid, 1.32 g/L NaCl, 0.3% bisacrylamide, 0.05 TEMED, 0.08% ammonium persulfate doped with 0.45% NaCl and deionized water) to resemble human soft tissue permittivity and conductivity at 64 MHz [41]. Fiber optic temperature probes (FISO Technologies, Quebec, Canada) were placed next to each distal solenoid loop coil, to the junction point of the catheter loopless channel whip, and to a remote position in the gel phantom. Real time SSFP used the following parameters: TR/TE, 3.67/1.84 ms; slice thickness, 6 mm; flip angle, 45°; FOV, 330 × 248 mm; bandwidth, 700 Hz/px. The phantom was centered on the scanner table and magnet isocenter set to middle.

Temperature measurements at different insertion length parameters were conducted first to determine the critical insertion length in the bore center and next at this critical insertion length progressively away from the isocenter horizontally.

## Results

### Flexibility test

Shaft stiffness was directly related to braiding composition stiffness. When the number of nitinol wires is reduced in the composite, the flexibility increases in proportion (Figure 2c). The commercial braided (stainless steel) 7 Fr Cook *Shuttle *introducer sheath stiffness was comparatively greater than all tested braiding configurations. Even though the catheter shaft has two coaxial braiding layers, the shaft rigidity was still lower than the commercially available *Shuttle *introducer.

### Torquability test

Shaft torque transmission also is related to the stiffness of the braiding layer. As the cooper wire proportion increases in the braiding layer, shaft torque transmission decreases. The 7 Fr steel *Shuttle *introducer sheath showed better torque response.

Flexibility and torquability of the catheter shaft that has a 12:4 (NiTi:Cu) braiding wire ratio were similar to the catheter shaft that has an 8:8 (NiTi:Cu) ratio. Therefore, because copper has superior conductivity, we chose the 8:8 (NiTi:Cu) ratio for all subsequent experiments.

### Visibility performance results

**Figure 5:**
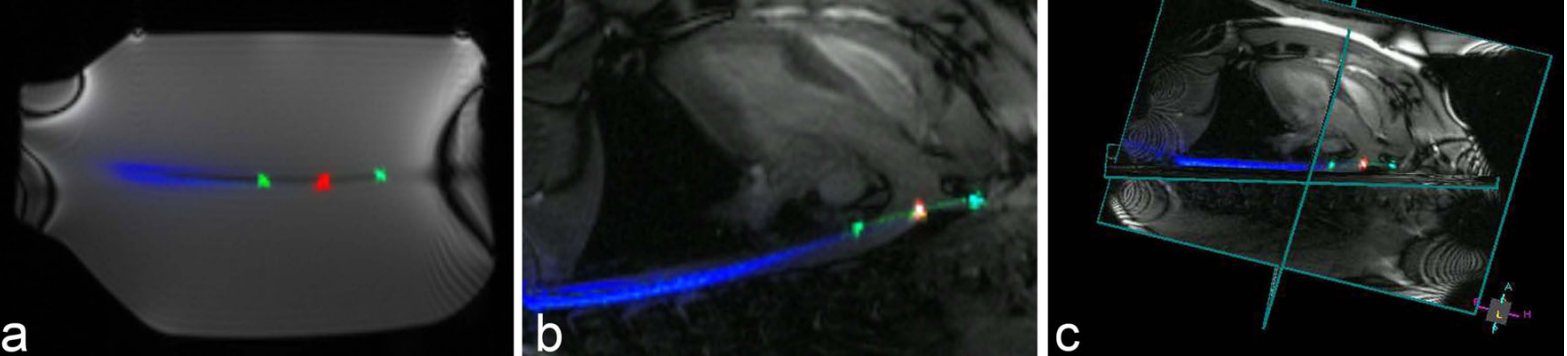
***in vitro *and *in vivo *visibility performance**. **(a) **Phantom MR image acquired with three channel catheter. **(b) **The active catheter was inserted percutaneously from the femoral artery, through the aorta, and into the left subclavian artery. **(c) **Multi-slice volume-rendered real-time MR of procedure described in panel (b) depicting the anatomic context. Device-related signal is evident in all slices. Independent catheter receiver channels allow colorized reconstruction of the tip (first (arrow) and third coil, green; middle coil, red; catheter shaft, blue).

The distal loop channels and the entire catheter shaft (70 cm) were conspicuous relative to background during both in vitro and in vivo experiments (Figure 5). The catheter shaft showed good longitudinal signal homogeneity while it was advanced through the phantom or the body.

**Figure 6:**
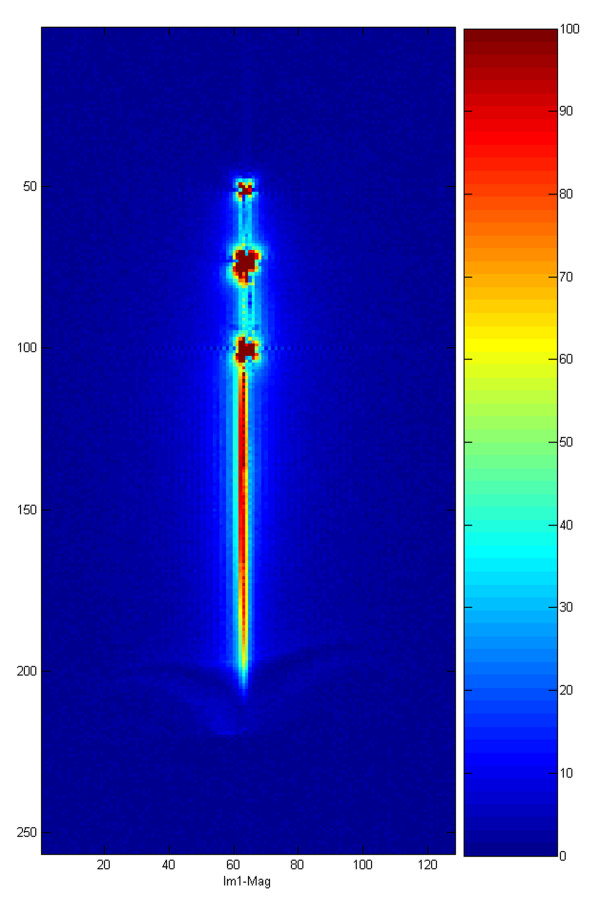
**Signal-to-noise ratio profile of the active laser catheter is mapped in normalized SNR units**.

The SNR profile map of the entire system (Figure 6) shows that the sensitivities of the loopless (shaft) antenna and distal (loop) antennas are similar, and sufficient to distinguish distal shaft from background.

### Heating test results

**Figure 7:**
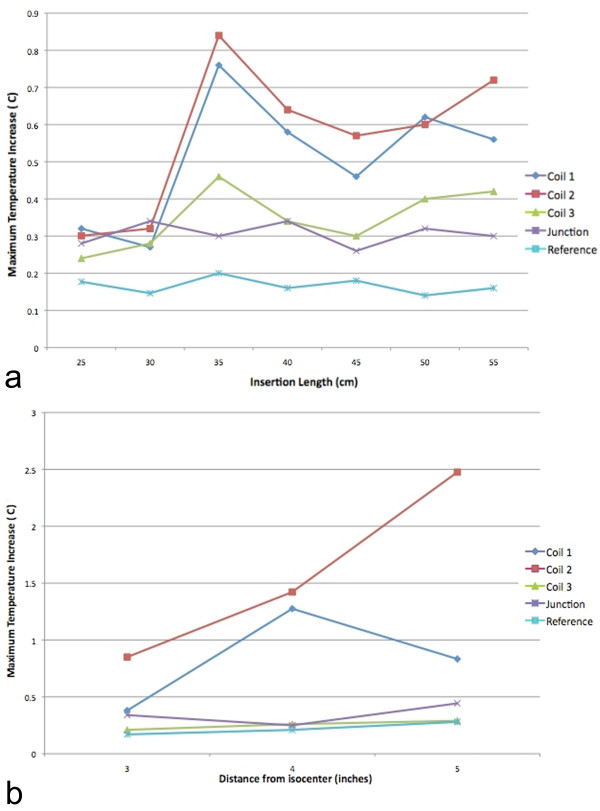
**The heating test results**. **(a) **maximum temperature rise versus insertion length graph when the active catheter placed to the isocenter. **(b) **maximum temperature rise measurements for different horizontal offset position relative to isocenter.

The second distal tip coil heated the most. Insertion-length-dependent temperature rises were all lower than 1°C. The critical insertion length was 35 cm, beyond which further insertion was associated with less heating. The maximum temperature rise was observed as 0.84°C for the critical 35 cm insertion length, at the second distal tip coil (Figure 7a). The temperature increase values were below 2°C within 10 cm horizontal offset from isocenter, but reached a maximum 2.48°C at the critical insertion length when positioned 12.5 cm horizontal from isocenter (Figure 7b). This horizontal position is only 2.5 cm from the lateral wall of the phantom.

## Discussion

We report the design and testing of a clinical grade active catheter for interventional MR that combines suitable mechanical performance with MR visibility. Specifically, we manipulated the braiding and polymer materials to serve both mechanically and electrically as a loopless antenna, to address the clinical requirement for whole-shaft visibility. In addition, we engineered this system to accommodate multiple transmission lines that we used for distal microcoils to indicate distal catheter position and orientation.

Loopless antennae are attractive candidates for whole-shaft MR catheter visibility because of relatively homogeneous signal profile and high near-field sensitivity. We used braided wires as conductors and polymers as insulators because of their mechanical properties. However the spacing between braided wires attenuates antenna signal. To address this, we tuned the whip length to minimize the antenna input resistance and optimize SNR [33].

Interventional catheters commonly incorporate steel braiding which is incompatible with MR. Nitinol has low electrical conductivity, σ (1 × 10^6 ^S/m) but great flexibility and kink resistance. Copper, which has higher electrical conductivity (σ = 5.8 × 10^7 ^S/m but lower strength and torque response. We combined the two in composite braids and empirically determined an optimum braiding configuration of nitinol:copper 8:8. The resulting design is less stiff than a similar size commercial steel introducer sheath system as expected, but highly conspicuous for interventional MR. Alternatively, we could have plated our nitinol braids with highly conductive metal, such as silver, σ = 6.28 × 10^7 ^S/m, or gold, σ = 4.26 × 10^7 ^S/m to exploit the skin effect of RF transmission and retain the mechanical properties of nitinol.

In order to provide the desired mechanical properties within the noted dimensional constraints, some catheters incorporate dual inner- and outer-braided wires. Each is typically embedded in a polymer to impart desired mechanical properties. The inner braided wire system is typically the primary source of compression resistance. Compression resistance allows the catheter to advance through the vessel against friction without undue axial compression ("accordion damage") of the shaft. Compression resistance also affords tactile feedback to the operator. By contrast, the outer braiding typically imparts torque transmission because of its larger moment-arm relative to the shaft axis. During catheter navigation, friction may store undesired torque energy that may release in spring-like fashion with unintended consequences.

Overall, our active guiding catheters have similar mechanical performance characteristics to X-ray devices used for similar applications, albeit at the expense of thicker walls because of additional engineering requirements. Nevertheless we have shown it is possible to manufacture active catheters with acceptable rigidity that is comparable to conventional X-ray devices. Our catheter shaft was assembled to form a tapered transition between the catheter whip and double-braided shaft to provide smooth and strong transitions between distal and proximal part of the device.

Long conductive structures such as transmission line and metal braiding layer may heat during MR [42]. We used braiding layers as components of a loopless antenna and we used a positive-intrinsic-negative (PIN) diode in decoupling circuits to reduce heating due to amplification of the electric field around the catheter conductors during RF transmission. The PIN diode shorts out the connector end of the antenna during MR excitation when a positive direct current voltage is supplied by the scanner. The short circuit is transformed to high impedance at the antenna junction to reduce current and heating. During reception, the diode is biased off, and the MR signal is conducted from the catheter antenna channels to the scanner. Preliminary heating tests were performed to evaluate the detuning circuit performance for different insertion lengths by using same real time SSFP sequence that is used for real time MR. All temperature rise measurement values were lower than the FDA recommendations [43] until the catheter was positioned 12.5 cm away (2.5 cm away from the phantom edge) from the isocenter. However, because of the complexity of the heating phenomena during MR, more comprehensive heating tests must be performed.

## Conclusion

We developed a conspicuous clinical-grade 7Fr active catheter by exploiting a novel (8:8 copper:nitinol) braiding configuration that serves as a loopless antenna in a polymer extrusion. The system incorporates multiple transmission lines for additional three distal tip microcoil channels. As a result, the catheter enjoys both whole-shaft and distal-tip visibility during real-time MR, and satisfactory flexibility and torquability for proposed clinical interventional procedures. Preliminary heating tests showed that temperature rise values along the catheter shaft were lower then 2°C until the catheter was positioned 12.5 cm away from the isocenter and 2.5 cm away from the phantom edge. This compact design promises wide applicability for interventional cardiovascular MR devices.

## Competing interests

OK is an inventor of patent applications for polymer shaft visualization design assigned to the National Institutes of Health. No other financial conflicts of interest are identified.

## Authors' contributions

OK and RJL drafted the initial article, OK and CES performed the literature search, OK, RJL, CO, ERM, CES, AZF, MAG and KR contributed the article content and participated in editing and final drafting of the manuscript. All authors read and approved the final manuscript.
